# Evaluating the Use of Virtual Reality in Medical School Neuroanatomy Curriculum: A Nonrandomized Pre–Post-Intervention Study with Medical Students

**DOI:** 10.1089/jmedxr.2024.0036

**Published:** 2025-04-11

**Authors:** Shannon K.T. Bailey, Antonious Y. Rizkalla, Ernesto A. Mejias Vazquez, Henry E. Blagden, Aditya Garg, Colleen C. Reiner, Srinivas Bharadwaj

**Affiliations:** ^1^ Center for Advanced Medical Learning and Simulation, USF Health, Tampa, FL, USA.; ^2^ Department of Medical Education, Morsani College of Medicine, University of South Florida, Tampa, Tampa, FL, USA.; ^3^ Department of Molecular Biosciences, University of South Florida, FL, USA.; ^4^ Department of Chemistry, University of South Florida, FL, USA.

**Keywords:** medical school, neuroanatomy, spatial ability, virtual reality

## Abstract

The purpose of this study was to examine the effectiveness of including a 1–1.5 h virtual reality (VR) lesson in the neuroanatomy curriculum for first-year medical students. We examined post-VR knowledge of neuroanatomy as our primary outcome measured by neuroanatomy tests, and students’ subjective experience of VR as our secondary outcome measured by the System Usability Scale (SUS) and a questionnaire. Neuroanatomy presents a significant challenge for students to learn due to the complexity of brain structures and their spatial relationships. VR offers an immersive platform that could aid in understanding three-dimensional (3D) structures. This study (pre–post-intervention without control) included 12 medical students enrolled in a first-year neuroanatomy course. Participants completed a neuroanatomy knowledge pre-test, then participated in a VR-based neuroanatomy lesson (intervention), followed by a neuroanatomy knowledge post-test using the same content created by the neuroanatomy course instructor. Our intervention was a VR lesson with a narrated walk-through of high-resolution 3D volumetric renderings of brain anatomy, focusing on cortical regions, subcortical structures, and major arteries. Students could pause the narration and manipulate 3D images (i.e., rotate, zoom, and bisect). Percent correct on the neuroanatomy post-test (*M* = 66.67%, *SD* = 12.85%) improved significantly from the pre-test (*M* = 52.08%, *SD* = 14.99%, *p* = 0.004, and *d* = 1.038). Student subjective experience using VR was rated below the benchmark on the SUS. Most rated their subjective experiences of the VR intervention positively (above the midpoint) on questionnaire items. Furthermore, students completed a validated measure of spatial ability (Paper Folding Test), and exploratory analysis revealed that students with lower spatial skills may benefit from the VR intervention and perform comparably to their peers who have higher spatial skills. Results indicated our VR neuroanatomy lesson may be associated with enhanced neuroanatomy knowledge among first-year medical students who have lower spatial skills. Further studies are needed to optimize the effectiveness of implementing VR in neuroanatomy education.

## Introduction

Medical schools are increasingly adopting immersive virtual reality (VR) to practice clinical and communication skills,^1^ and VR may also be useful for challenging topics in preclinical curricula related to complex spatial understanding. VR may provide benefits for medical education over traditional education materials by providing both immersion and interactivity relevant to the learning content. This study explored the effectiveness of incorporating a novel VR lesson into the neuroanatomy course for first-year medical students. Neuroanatomy is a difficult subject for medical students (and other health professional students) due to the complex geometry of the brain. Many key structures are small and are organized around non-geometric surface contours and internal fluid spaces called ventricles. The narrated VR lesson guided students through brain structures and spatial relationships and asked students to manipulate the high-resolution volumetric renderings (i.e., rotating structures, and bisecting planes). Prior research supports the idea that manipulating three-dimensional (3D) visualizations in VR may provide additional visuospatial information useful for understanding anatomy.^2^ This study sought to investigate the effectiveness of integrating VR content into the medical school curriculum, assessing the primary outcome of learning neuroanatomy knowledge and the secondary outcome of student experience with the novel VR technology. The learning outcome of neuroanatomy knowledge was measured quantitatively using a pre- and post-test of neuroanatomy from the course instructor. We hypothesized that students would improve significantly between neuroanatomy knowledge pre- and post-tests. Student experiences with the VR technology were measured quantitatively using a validated measure of usability (i.e., System Usability Scale [SUS]) and a Subjective Experience Questionnaire that included Likert-type survey items. We hypothesized that students would rate usability scores above the benchmark on the SUS (>68 on a 100-point scale) and questionnaire items above the midpoint of the scale (>3 on a 5-point scale). Students were also asked to provide written responses about their experiences with the VR technology, which were analyzed qualitatively for response themes. Furthermore, this study explored the potential relationship between spatial ability (Paper Folding Test; [PFT]) and the primary outcome of improved neuroanatomy knowledge post-VR intervention in an exploratory analysis. We hypothesized that the VR intervention would help students with lower spatial ability perform at the level of individuals with higher spatial ability on the post-test of neuroanatomy knowledge. Implications for the logistical considerations of implementing VR in course labs are discussed.

### Evidence for VR in education

There is growing evidence that VR in healthcare education is beneficial for learning, yet results vary based on many factors: Achievement of learning outcomes from VR educational interventions depends on the type of task being trained (e.g., procedural, decision-making), the type of VR being implemented (e.g., 3D animated environment, 360° video), the usability of the VR system, the learner’s level of knowledge and individual characteristics (e.g., student, resident, and healthcare professional), among many other factors.^1,3–7^ For example, a scoping review published in 2023 found that there were over 60 commercially available VR applications for healthcare education, and each may have specific benefits and limitations for different intended learning outcomes.^5^ Another systematic review of VR in medical education found that most content fit into one of five categories: anatomical knowledge, procedural skills, surgical procedures, communication, or clinical decision-making.^1^ The review found evidence that learning basic anatomical knowledge in VR was effective (i.e., learning outcomes were achieved) but not necessarily better than other methods (e.g., textbook, tablet, augmented reality materials). Evidence for the other categories was inconclusive (i.e., procedural skills, communication, and clinical decision-making), highlighting the need for more primary studies of VR in medical education with more consistent use of reporting guidelines.

### Theoretical background

The effectiveness of VR may vary depending on the specific learning objectives, as different features of VR technology can be more or less beneficial for specific tasks. There are several potential explanations for why VR may be useful for medical education compared to traditional methods. From a cognitive psychology perspective, researchers have explored the characteristics of VR training that impact learning outcomes, focusing on the roles of immersion and interactivity as critical factors that affect cognitive processing.^8^ For example, VR may benefit neuroanatomy education by offering detailed, 3D visualizations that more accurately represent the real human complex spatial relationships between brain structures. This immersive approach in VR may provide richer visuospatial information compared to using flat 2D displays (e.g., computer screens, classroom projectors) to present 3D structures, which are typical in neuroanatomy courses. Interactivity in VR - that is, manipulating objects in a virtual environment (e.g., rotate, enlarge, bisect) - may also play a role in enhancing neuroanatomy education, because a learner manipulating 3D objects can improve their understanding of spatial information compared to either passive viewing of 3D information without manipulation or interacting with 2D virtual objects.^2,9^ According to embodiment theory, interactivity in the VR environment provides a learning experience in which learners encode both the physical movement of interacting with an object and visual information, creating a multi-sensory representation of that information in their memory.^10^ Furthermore, the impact of VR on learning outcomes may depend on learner characteristics, such as level of education or spatial ability.^8,11^ The current study explored the possibility of a relationship between a learner’s spatial ability and their experience with the VR lesson because preliminary evidence has shown that VR training was able to help learners with lower spatial ability to perform comparably to those with higher spatial ability.^8^ The researchers theorized that VR provides more visuospatial information (immersion) and object manipulation (interactivity) than 2D learning materials provide, supporting learners with lower spatial ability who might benefit from more information and active processing.

### Implementing VR in medical education

In a scoping review of VR integration in medical schools, Mergen et al. identified common advantages and disadvantages of utilizing VR.^6^ Mergen et al. concluded that the advantages over other types of training included accessibility and convenience, practicing skills in a safe environment, variability and repeatability of content, and automatic assessment and feedback. Disadvantages were costs, technical limitations, user experience issues, as well as inconsistent empirical evidence on how to implement VR in healthcare education. Evidence is still emerging around how to implement VR in medical schools and healthcare education programs, yet the reported experiences of students and educators using VR are mostly positive. A recent systematic review of VR in medical education found that in all but one study, participants rated VR more highly on subjective learning experience than other training methods.^1^ For example, a study found that medical students rated VR as a more acceptable format than computer-based simulation for learning airway intubation (*d* = 0.66).^12^ Another scoping review found an overall positive perception of integrating VR into medical education by medical students and faculty; however, appropriate methods for integration into curricula were unclear as standards and validated guidelines are currently lacking.^6^ Although there is limited research on implementation strategies, considerations for incorporating VR in medical school curricula have been suggested. These include taking into consideration cost, available resources (e.g., staff and space), scalability (e.g., how many students can participate at a time), interoperability (e.g., integration with other simulation technology), and whether VR is the appropriate format for the intended learning outcomes.^13^

## Method

The current study utilized the approach of providing medical students with an optional educational opportunity in the form of a novel VR lesson related to their current preclinical course curriculum during dedicated lab hours. The study was approved by the university’s institutional review board and was conducted in April 2024 over 2 weeks. The Transparent Reporting of Evaluations with Nonrandomized Designs reporting guideline is used to report the study.^14^

### Objectives

The primary objective of the study was to determine if a VR educational intervention in a medical student neuroanatomy course results in improved neuroanatomy test scores on understanding spatially complex human brain structures. We hypothesized that students would improve significantly between neuroanatomy knowledge pre- and post-tests. The secondary objectives were to assess the usability and subjective experience of integrating VR technology into a neuroanatomy course to address critical gaps in understanding VR’s feasibility for inclusion in the medical education curriculum. We hypothesized that students would rate usability scores above the benchmark on the SUS,^15^ (>68 on a 100-point scale), and questionnaire items above the midpoint of the scale (>3 on a 5-point scale). Exploratory objectives included assessing the relationship between an individual’s spatial ability, neuroanatomy test performance, and experience with the VR educational intervention. We conducted ancillary statistical analyses to investigate this relationship (i.e., bivariate correlation, analysis of covariance [ANCOVA]).

### Outcomes

The measures are listed in Table 1. All measures were collected on a computer using Qualtrics survey software. All measures except the pre-test of neuroanatomy knowledge were administered after the VR lesson.

**Table 1.** Outcome Measures

Measure name	Type	Number of items	Description
Neuroanatomy test	Content knowledge (Quantitative)	20	Pre- and post-test of neuroanatomy content knowledge created by the neuroanatomy course instructor including orientation of brain surface features, cortical regions, relationship of structures, major arteries, and brain regions they perfuse. Items were multiple choice and fill-in-the-blank format. Scores are reported as percentages correct (out of 20 total items).
System usability scale (SUS; Brooke, 1996)^15^	Usability (Quantitative)	10	A validated and reliable post-intervention measure of a technology’s usability using Likert-type items. Scores above 68 on the 100-point scale are considered “Good” usability.
Subjective experience questionnaire	Reactions (Quantitative; Qualitative)	10	Post-intervention questionnaire of experience with the VR technology and intent to use in the future, including 8 Likert-type items (Table 3) and 2 written responses on usability (see *Written response* section).
Paper folding test (PFT; Ekstrom et al., 1976)^16^	Spatial ability (Quantitative)	10	A validated and reliable timed (3 min) measure of spatial visualization ability in which a participant must match a folded piece of paper with a hole punched through to an unfolded piece of paper with the corresponding hole punch. Calculated scores range between 0 and 10, with higher scores indicating higher spatial ability.

VR, virtual reality.

### Participants

Medical students were recruited from a first-year neuroanatomy course in the United States (*n* = 12; Age: *M* = 24.58, *SD* = 1.17; Sex: 3 females, 9 males) via a course announcement and self-selection. Prior to participating in the study, participants signed an informed consent form. A single group pre/post-test design was utilized, so all participants were assigned to the VR education intervention. For the main outcome variable of pre- and post-test neuroanatomy knowledge, an *a priori* power analysis was conducted using G*Power (version 3.1.9.7) for a dependent sample *t-*test (one-tailed) at α = 0.05, 80% power, and large effect size (*Cohen’s d* = 0.80), which indicated a total sample size of 12 participants needed. Students completed the VR educational intervention individually in a lab setting at their medical school’s simulation center during their course’s dedicated lab hours on a single day. Inclusion criteria included being a current medical student enrolled in the neuroanatomy course and at least 18 years of age. Exclusion criteria included individuals who were not medical students, were less than 18 years of age, were colorblind or with uncorrected vision (including those lacking stereoscopy, severe amblyopia, or other eye disorders), or required unique accommodation. Students were not provided with incentives to participate.

### Study design

**FIG. 1.**
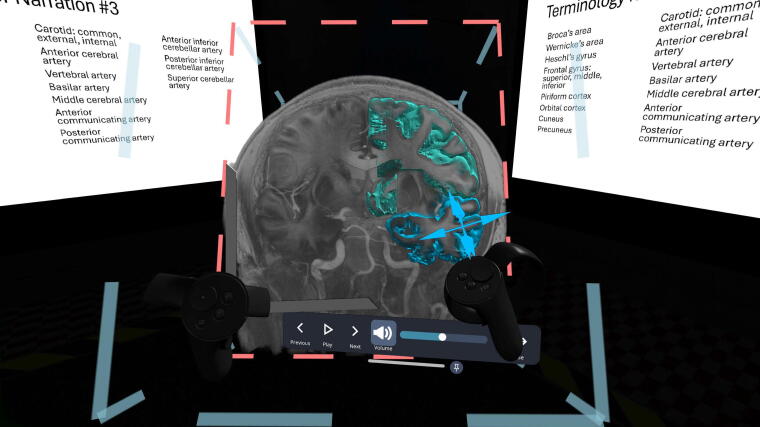
Screenshot of VR neuroanatomy lesson. VR, virtual reality.

The study design was a single-group, pre- and post-intervention (nonrandomized, repeated measures) study of a VR educational intervention for neuroanatomy knowledge. Students completed a pre- and post-test of neuroanatomy knowledge. Between the pre- and post-test, students were seated in a lab where they used a VR headset (Meta Quest 2) to interact with VR software (syGlass; Fig. 1; see Supplementary Video S1 in *Supplementary Digital Content*) that provided a 3D volumetric rendering of a whole-head MRI, with vascular contrast, that revealed anatomical brain structures along with recorded narrations by a professor in the medical school who holds a Ph.D. in Neuroscience. Students were able to manipulate the high-resolution 3D volumetric renderings, including bisecting planes as instructed by the narrator. The VR content included three narrations with interactive volumetric brain structures, with each lesson ranging from 15–20 min in length. The topics corresponded with the neuroanatomy course curriculum and included: 1. Orientation to brain surface features, cortical regions, and the relationship of the midbrain to subcortical structures; 2. Basal ganglia, hypothalamus, limbic and midbrain structures, and their spatial relationships to each other; and 3. Major arteries and the brain regions that they perfuse. The VR lesson was delivered via cloud rendering over Wi-Fi internet such that the headsets were untethered (i.e., not attached to a computer via a cord).

**FIG. 2.**
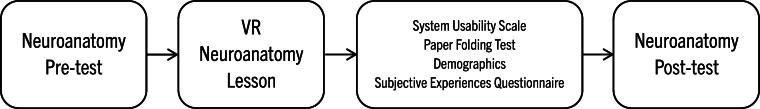
Participant flow diagram.

Student interactions with the VR lesson lasted between 1 and 1.5 h total. The flow of the study is presented in Figure 2. Following the VR lesson, students completed the SUS,^15^ PFT,^16^ subjective experience questionnaire, and the post-test of neuroanatomy knowledge. Participant demographics were also collected including participant characteristics and self-reported previous participation in simulations related to the neuroanatomy content. All measures were completed on the computer using participant ID numbers to avoid participant identification by researchers or the course instructor.

### Statistical analyses

The statistical analyses were conducted using IBM SPSS 29, with α < 0.05 (two-tailed) unless otherwise specified. Correlations of measures were analyzed using Pearson *r* bivariate correlation at α < 0.05 (Table 2). For the primary outcome measure of pre- and post-test neuroanatomical knowledge (Neuroanatomy Test), a dependent sample *t-*test was conducted. For the secondary outcome variables, descriptive statistics are reported for the SUS and all subjective experience questionnaire items. For the exploratory variable of spatial ability, an ancillary statistical analysis was conducted to investigate the relationship between spatial ability (i.e., PFT) and learning outcome (Neuroanatomy Test) using repeated measures ANCOVA controlling for participant PFT score. Twelve participants completed the study and were included in the analyses, and there were no other protocol deviations. Participants with missing data were excluded from individual analyses (see reported *n* for each statistic). Adverse events include one participant reported feeling slight dizziness (i.e., simulator sickness).

**Table 2.** Pearson *r* Bivariate Correlation of Measures

	Pre-test % correct	Post-test % correct	Normalized gain score	SUS
Pre-test % correct	—			
Post-test % correct	0.499	—		
Normalized gain score	−0.589*	0.406	—	
SUS	−0.182	−0.090	0.098	—
PFT (*n* = 11)	0.755**	−0.020	−0.783**	−0.391

*Note*. *p < 0.05; **p < 0.01; *n* = 12, except for correlations with PFT, where *n* = 11.

PFT, paper folding test; SUS, system usability scale.

## Results

### Correlation of measures

#### Primary outcome

The hypothesis that students would score better on the neuroanatomy post-test than the pre-test following the VR lesson was supported. The difference between neuroanatomy pre- and post-test scores was significant with a large effect size (paired *t*[11] = −3.594, *p* = 0.004, *d* = 1.038). The average improvement between pre-test (*M* = 52.08%, *SD* = 14.99%) and post-test (*M* = 66.67%, *SD* = 12.85%) was 14.58% (*SD* = 14.05%, *95% CI* = [5.65%, 23.51%]), and the normalized gain score average was 0.03 (*SD* = 0.03). The normalized gain score is conceptualized as the amount of improvement possible given a student’s pre-test score and is calculated using the formula:

$$\frac{\;(\mathrm{posttest}\;-\;\mathrm{pretest})\;}{\left(100\text{\% }-\;\mathrm{pretest}\right)}$$

#### Secondary outcomes

Counter to our hypothesis, the average SUS score was less than the benchmark score of 68 (*M* = 51.46, *SD* = 14.16, *95% CI* = [42.46, 60.46]). Supporting our hypothesis, the subjective experience items were rated positively (above the midpoint) on Likert-type scales (Table 3). Students somewhat or strongly agreed that the VR lesson was clear and relevant to their neuroanatomy course (92%), that the VR lesson improved their performance on the content (75%), and that they would intend to use the VR lesson if available to them (58%).

**Table 3.** Subjective Experience and Intent to Use Survey Results

Item	M	SD	95% CI lower	95% CI upper
1. This training helped me to learn the information more quickly. (1 = strongly disagree, 5 = strongly agree)	3.75	0.97	3.14	4.36
2. The training information provided with the application is clear and relevant to the course content. (1 = strongly disagree, 5 = strongly agree)	4.33	1.15	3.60	5.07
3. This training helped to improve my performance on the content. (1 = strongly disagree, 5 = strongly agree)	3.83	0.83	3.30	4.36
4. If it were available to me, I would use this training application going forward. (1 = strongly disagree, 5 = strongly agree)	3.67	1.15	2.93	4.40
5. Using this training application going forward would allow me to learn new content more quickly. (1 = strongly disagree, 5 = strongly agree)	3.83	1.19	3.08	4.59
6. If it were available to me, I would prefer this training application compared to more traditional styles of training. (1 = strongly disagree, 5 = strongly agree)	3.42	1.38	2.54	4.29
7. I will seek out opportunities to learn via this training application in the future. (1 = strongly disagree, 5 = strongly agree)	3.67	1.30	2.84	4.49
8. If virtual reality simulations were made available to you, how likely are you to use them voluntarily? (1 = extremely unlikely, 5 = extremely likely)	3.83	1.19	3.08	4.59

*Note: n* = 12.

##### Written responses

To better understand medical students’ views of integrating VR into the medical school curriculum, participants responded to the free text question: *What would make you more likely to use VR simulations?* Two independent reviewers coded the written statements for themes. After agreement was made by the two reviewers, one of the study authors (SB) tallied the frequency for each type of response (i.e., the comfort of a headset, reduced glitches) and consolidated the responses (Table 4). The responses highlight several key themes for increasing the likelihood of using VR simulations. The major factors influencing VR usage include headset comfort, integration into schedules, improved quality and customization, better feedback mechanisms, and reduced technical issues.

**Table 4.** Factors Influencing Intent to Use VR

Theme	Frequency	Description
1. Comfort and usability	5	Comfortable headsets: 3 Ease of use: 2
2. Integration and accessibility	4	Integrated into the curriculum: 2 Ease of access: 2
3. Quality and customization	4	Better quality: 2 Customizable experience: 2
4. Feedback and interactivity	2	Improved feedback: 1 Interactive features: 1
5. Technical issues	2	Reduce glitches: 1 Non-narrator version: 1

VR, virtual reality.

Using the same methodology, the second free text item asked participants whether they had comments specific to the usability of the VR system. The responses indicated major factors influencing VR user experience included the need for improved comfort and ergonomics, better usability and navigation with tutorials, fewer technical issues, and enhanced customization options. Students appreciated certain helpful features, such as the bisecting tool. The responses highlight several key themes for enhancing the use of VR systems (Table 5).

**Table 5.** Factors Influencing VR Experience

Theme	Frequency	Description
1. Usability and navigation	9	Tutorial needed: 5 Understanding controls: 4
2. Ergonomics	6	Uncomfortable headset: 4 Eye strain: 2
3. Customization	6	Ability to adjust distance/speed: 2 Ability to view structures independently: 1 Customization issues: 3
4. Technical issues	4	Crashes: 3 Slow load times: 1
5. Positive features	3	Helpful tools: 3

VR, virtual reality.

### Ancillary analysis

An exploratory outcome was the relationship between spatial ability (PFT) and learning outcome (Neuroanatomy Test). PFT scores can range from 0–10, with higher scores indicating more correct responses. Participant PFT scores in this study ranged from 3–10 (M = 7.69, *SD* = 2.02), with a skew toward higher scores. As reported in Table 2, spatial ability (PFT) was not correlated significantly with usability ratings (SUS); however, spatial ability (PFT) was positively correlated with pre-test scores on the neuroanatomy test (*r* = 0.755, *p* = 0.007). To account for individual differences in spatial ability (PFT) on the baseline scores of the neuroanatomy test, repeated measures ANCOVA were conducted on pre- and post-test neuroanatomy scores with PFT as a covariate. Mauchly’s Test of Sphericity was not significant (*p* > 0.05), so no corrections were made. There was a significant difference between pre- and post-test neuroanatomy test scores, with higher scores following the VR lesson (*F* [1,9] = 26.53, *p* < 0.001, ηp^2^ = 0.747). There was also a significant interaction effect of neuroanatomy test scores with spatial ability, where PFT was significantly related to neuroanatomy pre-test scores, but not neuroanatomy post-test scores (*F* [1,9] = 16.305, *p* = 0.003, ηp^2^ = 0.644), indicating that participants performed better on the neuroanatomy post-test regardless of spatial ability (PFT). This result provides exploratory evidence that the VR lesson may improve neuroanatomy test scores for those with lower spatial ability (PFT) to perform at the level of those with higher spatial ability on the neuroanatomy post-test.

## Discussion

This study investigated the feasibility of integrating a novel VR lesson into neuroanatomy education for first-year medical students, aiming to enhance their understanding of brain structures and spatial relationships. For our primary outcome of improved test scores on understanding spatially complex human brain structures, our hypothesis that student performance on the neuroanatomy test would improve following the VR lesson was supported, indicating that VR is a promising tool for conveying complex spatial information in neuroanatomy.

The secondary outcome focused on the usability and subjective experience of incorporating a novel VR technology into the medical school curriculum. We hypothesized that students would rate the usability and user experience of the VR lesson positively on the SUS and items on a subjective user experience questionnaire. This hypothesis was partially supported: while most students found the VR lessons engaging and user-friendly as indicated by the above mid-point ratings on the subjective user experience questionnaire items, a subset reported difficulties with the technology in the written responses, such as simulator sickness or challenges navigating the VR environment. Although our findings demonstrated significant improvements in post-test neuroanatomy test scores following VR lessons, the below-average SUS usability rating suggests developments could be made to improve students’ experience in this VR system. Conversely, students rated their subjective experience positively (above the midpoint) on the subjective user experience questionnaire items, indicating the perceived benefits of using VR and intent to use VR for training in the future. These results underscore the potential of VR for medical education but also highlight the need for further refinement to optimize usability. These results suggest that while VR has potential, further technology modification and user training may be necessary to optimize VR learning experiences for all learners. Based on the written responses, students suggested modifications to the novel VR system that could be useful for other VR lessons. For example, students indicated the navigation and user interface could better reflect user expectations for more intuitive controls and desired customization of controls, such as changing the narration speed. Students also wanted more feedback on the interactive portions of the lesson with details of what they did correctly or incorrectly. Finally, students suggested having additional lab or class time dedicated to learning to use VR technology and more self-paced VR content.

Exploratory analyses examined the relationship between an individual’s spatial ability (PFT) and neuroanatomy test scores following the VR intervention. Consistent with prior research, our findings indicated that VR benefited students with lower spatial ability, helping them perform at a level comparable to those with higher spatial ability in this study.

The feasibility of implementing the VR intervention in a medical school curriculum was generally promising, though several challenges were identified. The implementation faced barriers, including technical issues, which occasionally disrupted the learning experience. Because the VR lesson was rendered in the cloud and delivered via a Wi-Fi internet connection, fluctuations in internet speeds and multiple headsets connected to the limited internet bandwidth affected the stability of the connection, causing system lags or crashes. In addition, one student experienced simulator sickness, which indicates VR may be of limited utility for some individuals. Moreover, logistical considerations, such as the availability of VR equipment during dedicated lab hours and the need for technical support, were identified as significant factors that could influence the scalability of VR integration into broader medical education programs. Students in this study participated during dedicated lab time for the course, which meant little time was available for additional training or practice using the new system. Implications for medical education curricula include considering both the educational benefits and usability factors when integrating VR technology into courses.

### Limitations and future research

Although this study contributes to the expanding body of research on the use of VR in medical education, its findings should be interpreted in light of study limitations. The study was a pilot investigation involving a small sample of 12 medical students in a single course, on a single day, which limits the generalizability of the results to broader contexts. In addition, the use of a single group, nonrandomized design means that learning outcomes were not compared to those of a conventional coursework control group. As a result, no conclusions can be drawn about how VR compares to conventional coursework. Finally, a 14% improvement in neuroanatomy test scores between pre- and post-test is modest and may have been a result of a practice effect where students performed better on the post-test as a result of practice on the pre-test. Future research with larger, randomized samples and direct comparisons to traditional methods in a variety of courses will be necessary to assess the potential of VR in medical curricula more broadly.

## Conclusion

This pilot study demonstrates that a novel VR lesson can enhance neuroanatomy education by improving students’ understanding of spatially complex brain structures. Although the results indicate that VR may benefit students with lower spatial ability, thereby promoting more equitable learning outcomes, challenges such as technical issues and variability in user experience highlight the need for further refinement of this VR technology and its implementation in medical school curricula. Further, the usability issues with this VR lesson may not be reflective of other VR systems. Although the study’s limited sample size and design restrict broader generalization, the findings support continued exploration of VR as an educational tool in medical schools.

## Abbreviations Used

3D: Three-dimensional
ANCOVA: Analysis of Covariance
PFT: Paper Folding Test
SUS: System Usability Scale
VR: Virtual Reality
